# Association of Single Nucleotide Polymorphisms from Angiogenesis-Related Genes, *ANGPT2*, *TLR2* and *TLR9,* with Spontaneous Preterm Labor

**DOI:** 10.3390/cimb44070203

**Published:** 2022-06-30

**Authors:** Wioletta Izabela Wujcicka, Marian Kacerovsky, Adrian Krygier, Michał Krekora, Piotr Kaczmarek, Mariusz Grzesiak

**Affiliations:** 1 Scientific Laboratory of the Center of Medical Laboratory Diagnostics and Screening, Polish Mother’s Memorial Hospital-Research Institute, 93-338 Lodz, Poland; 2 Department of Obstetrics and Gynecology, University Hospital Hradec Kralove, Charles University, 500 03 Hradec Kralove, Czech Republic; marian.kacerovsky@gmail.com; 3 Biomedical Research Center, University Hospital Hradec Kralove, 500 03 Hradec Kralove, Czech Republic; 4 Laboratory of Molecular Diagnostics and Pharmacogenomics, Department of Pharmaceutical Biochemistry and Molecular Diagnostics, Medical University of Lodz, 90-151 Lodz, Poland; adriankrygier@o2.pl; 5 Department of Obstetrics and Gynecology, Polish Mother’s Memorial Hospital-Research Institute, 93-338 Lodz, Poland; krekoram@poczta.onet.pl; 6 Department of Gynecology and Obstetrics, Medical University of Lodz, 93-338 Lodz, Poland; mariusz.grzesiak@gmail.com; 7 Department of Gynecology, Reproduction and Fetal Therapy, and Diagnostics and Treatment of Infertility, Polish Mother’s Memorial Hospital-Research Institute, 93-338 Lodz, Poland; kaczmarekpiotr1@gmail.com; 8 Department of Perinatology, Obstetrics and Gynecology, Polish Mother’s Memorial Hospital-Research Institute, 93-338 Lodz, Poland

**Keywords:** spontaneous preterm labor, pregnancy, angiogenesis, genotyping, single nucleotide polymorphism, restriction fragment length polymorphism

## Abstract

In this study, we hypothesized that the changes localized at angiopoietin-2 (*ANGPT2*), granulocyte-macrophage colony-stimulating factor (*CSF2*), fms-related tyrosine kinase 1 (*FLT1*) and toll-like receptor (*TLR*) *2*, *TLR6* and *TLR9* genes were associated with spontaneous preterm labor (PTL), as well as with possible genetic alterations on PTL-related coagulation. This case-control genetic association study aimed to identify single nucleotide polymorphisms (SNPs) for the aforementioned genes, which are correlated with genetic risk or protection against PTL in Polish women. The study was conducted in 320 patients treated between 2016 and 2020, including 160 women with PTL and 160 term controls in labor. We found that *ANGPT2* rs3020221 AA homozygotes were significantly less common in PTL cases than in controls, especially after adjusting for activated partial thromboplastin time (APTT) and platelet (PLT) parameters. TC heterozygotes for *TLR2* rs3804099 were associated with PTL after correcting for anemia, vaginal bleeding, and history of threatened miscarriage or PTL. TC and CC genotypes in *TLR9* rs187084 were significantly less common in women with PTL, compared to the controls, after adjusting for bleeding and gestational diabetes. For the first time, it was shown that three polymorphisms—*ANGPT2* rs3020221, *TLR2* rs3804099 and *TLR9* rs187084 —were significantly associated with PTL, adjusted by pregnancy development influencing factors.

## 1. Introduction

Spontaneous preterm labor (PTL) is the leading cause of perinatal morbidity and mortality worldwide [1]. The greatest number of complications is observed in deliveries before the 34th week of gestation (2% of pregnancies) [2]. The incidence of respiratory distress syndrome, intraventricular hemorrhage, necrotizing enterocolitis and sepsis, as well as mortality, are inversely correlated with the gestational age at birth [3,4].

Among the PTL-related processes, an impaired placentation has been reported [5]. The placenta plays a key role for the proper development and survival of the fetus during pregnancy, supplying nutrients to the fetus and exchanging gases with its mother. The placenta also ensures an effective barrier against infectious agents [6,7]. Both vasculogenesis and angiogenesis are necessary to build a branched vascular network in placental villi [8,9]. A proper control of vascular and inflammatory processes is essential for placental development [10].

Various angiogenic factors, including vascular endothelial growth factor (VEGF), placental growth factor (PGF) and angiopoietin-2 (ANGPT2), are involved in the development of new blood vessels and vascular networks, while sFlt1, a soluble form of the VEGF receptor (sVEGFR1), demonstrates an opposite effect on VEGF and PGF [7,11]. In the case of ANGPT2, its significant anti-inflammatory function was also previously described [12]. In an experimental mouse model of preterm delivery (PTD), an administration of ANGPT2 shortened the time period to delivery, induced by LPS, through downregulation of TNF-alpha overproduction in maternal circulation, the placenta and fetal tissues, and by disrupting fetal angiogenesis associated with the loss of embryonic perfusion [13]. Considering the immune defense of the fetus, the expression of toll-like receptors (TLRs) 1–10 was determined in cytotrophoblast and syncytiotrophoblast placental cells, both timely and prematurely [14,15]. It was also observed that aortic angiogenesis was largely regulated by TLRs in response to injury [16]. In angiogenic cultures of rat aorta, the expression levels of TLR2, TLR4 and TLR8 were the highest after 24 h from injury and remained elevated during angiogenesis and vascular regression, while TLR5, TLR7 and TLR9 were consistently increased at the highest levels during vascular regression [16]. An induction of TLR2/6 by its agonist, the macrophage activating lipopeptide of 2 kDa (MALP2), was determined to promote angiogenesis mediated by the granulocyte-macrophage colony-stimulating factor (GM-CSF) [17,18]. In human pulmonary microvascular endothelial cells (HPMEC) and in smooth muscle cells (SMC) of pulmonary origin, the expression level of ANGPT2 increased significantly after TLR4 activation, while it was halved after a stimulation with the cytosine-phosphate-guanosine (CpG) ligand for TLR9 [19]. In ocular vascular diseases, the synthetic suppression of angiogenesis by CpG oligodeoxynucleotides (CpG-ODN) has been reported as TLR9-dependent for its absence in TLR9-deficient mice [20].

An array study of 50,000 gene-centric single nucleotide polymorphisms (SNPs) focused on 124 haplotype-tagging SNPs (tagSNPs) from 6 angiogenesis-related genes determined that *FLT1* rs12584067 and rs7335588 correlated with preeclampsia (PE) in African American women, while rs722503 polymorphism was more prevalent in Caucasian patients with the disease [21,22]. Considering *ANGPT2*, the rs3020221 polymorphism in exon 4 was suggested to influence gene or protein expression and inhibit vascular angiogenesis [23,24,25]. In the case of the *TLR2* gene, the synonymous rs3804099 polymorphism in the third exon of the gene has been associated with many infectious diseases, including bacterial meningitis and pulmonary tuberculosis (PTB) [26,27]. Regarding the *TLR6* gene, it has been observed that the non-synonymous rs5743810 polymorphism affects ligand recognition and reduces signal response, while the T allele decreases the signaling of nuclear factor kappa B (NF-kappa-B), associated with angiogenesis, in HEK293 cells, and increases plasma interferon gamma (IFN-gamma) levels [28,29,30]. Out of many *TLR9* SNPs, rs187084 and rs5743836, located at the promoter region, have been identified as the most important polymorphisms that affect gene transcription by regulating its promoter activity in Nawalma R20 B cells [31,32]. Regarding the *CSF2* gene, the rs25882 polymorphism is located in the exon region, which has been reported to influence the production of GM-CSF isoforms and the affinity between mRNA and ribosomes [33].

In addition to alterations in angiogenesis, PTL has also been correlated with the state of hypercoagulation [34]. Previously, an increase in procoagulant levels was found during pregnancy, along with a decrease in the intensity of anticoagulants and fibrinolysis [35,36]. It has been suggested that, in the third trimester, a lower platelet (PLT) count results from a greater consumption of PLTs in the uteroplacental unit [37]. As for PTL with intact membranes, higher thrombin production in the blood of pregnant women was determined, as it is associated with increased plasma levels of thrombin-antithrombin (TAT) complexes [38,39]. A study, conducted in women with premature uterine contractions, showed a significantly shorter prothrombin time (PT) and an activated partial thromboplastin time (APTT) in patients with PTL, compared to term deliveries [34]. Based on the significant contribution of ANGPT2 and GM-CSF factors, and sFlt1, TLR2, TLR6 and TLR9 receptors in angiogenesis, as well as in PTL and/or PTD, we hypothesized that the changes localized in the genes encoding those selected molecules were associated with PTL. We also hypothesized a possible effect of those genetic alterations on PTL-related coagulation. Therefore, we designed and set up a case-control genetic association study to examine the role of six selected SNPs from genes encoding ANGPT2, GM-CSF, sFlt1, TLR2, TLR6 and TLR9 molecules, in conjunction with APTT and PLT parameters, in susceptibility to PTL in Polish women. In this research, models of inheritance of polymorphisms in *ANGPT2* (rs3020221), *CSF2* (rs25882), *FLT1* (rs722503), *TLR2* (rs3804099), *TLR6* (rs5743810) and *TLR9* (rs187084) were analyzed to identify the genotypes of risk or protection against the disease.

## 2. Materials and Methods

### 2.1. Study Population

The study was carried out prospectively in 320 women with singleton pregnancies, hospitalized at the Department of Obstetrics, Perinatology and Gynecology, as well as at the Department of Obstetrics and Gynecology, of the Polish Mother’s Memorial Hospital-Research Institute (PMMH-RI) in Lodz, Poland, between August 2016 and March 2020. The population consisted of 160 women with spontaneous PTL with intact membranes and of the same number of term controls in labor (1:1 ratio, see Table 1). The patients, included both in PTL and the control group, were 18–40 years old. PTL was defined as regular uterine contractions on admission (confirmed by external tocometry) in combination with one of the following criteria: cervical dilatation ≥2 cm and/or cervical length <25 mm (as documented by digital examination and transvaginal sonography at enrollment or at sampling), and determined between 22 and 35 weeks of pregnancy. The control group of pregnant women were admitted to the Department for labor and delivery, from the 37th to the 41st week of gestation.

The exclusion criteria, both for PTL and the control groups of women comprised multiple pregnancy, congenital disorders, genetic syndrome, polyhydramnios, structural uterine defects, placenta previa, preterm prelabor rupture of membranes (pPROM), cervical insufficiency, the history of miscarriage, pregestational diabetes mellitus (DM) and fetal growth restriction (FGR). In addition, the pregnant women diagnosed with hypertension were excluded from the cohort of PTL cases. Blood pressure (BP) measurement and diagnosis of hypertension were performed according to the clinical practice guidelines of the Society of Obstetricians and Gynaecologists of Canada [40]. BP was measured in women who were seated with their arms at the heart level. We used a calibrated aneroid sphygmomanometer or an automated BP machine approved for use in preeclampsia. Hypertension was defined as systolic and diastolic BPs of ≥140 and ≥90 mm Hg, respectively, based on the mean of at least two measurements, taken at least 15 min apart, on the same arm. The study was approved by the Research Ethics Committee at the PMMH-RI (the approval number: 15/2019). Clinical samples were collected for diagnostic purposes and then anonymized for testing. Informed consent forms were signed by all the study participants in line with the recommendations of the Research Ethics Committee.

### 2.2. Blood Sample Processing

Peripheral venous blood samples were taken by puncture from the pregnant women on the day of admission. PLT parameters, including PLT count, PLT distribution width (PDW), the mean PLT volume (MPV) and plateletcrit (PCT) as part of complete blood count (CBC), were assayed, using the Fluorocell PLT reagent on a Sysmex XN-2000 automated hematology system (Sysmex, Kobe, Japan). APTT was determined using the HemosIL APTT-SP reagent on an ACL TOP 550 CTS automated system (Instrumentation Laboratory, Werfen Company, Bedford, MA, USA). Total DNA was extracted from 200 µL of whole blood samples using a Syngen Blood/Cell DNA Mini Kit (Syngen Biotech, Wroclaw, Poland).

### 2.3. SNP Selection and Genotyping

Six SNPs, localized in the genes encoding angiogenesis-related factors and TLRs, were selected according to the SNP database (dbSNP) of the National Center for Biotechnology Information (NCBI) [41]. Candidate polymorphisms were qualified into the study on the basis of: (1) The location in genes involved in angiogenesis and in PTL and/or PTD; (2) a high prevalence in the European population, with minor allele frequency (MAF) > 20%, provided by the NCBI Allele Frequency Aggregator (ALFA) project; and (3) a possible impact on the function of the encoded protein. All tested SNPs were genotyped by polymerase chain reaction-restriction fragment length polymorphism (PCR-RFLP) analysis, using the previously reported primers [25,42,43,44,45,46,47]. PCR products were digested using 10 U of appropriate endonucleases at specified enzyme temperatures for 16 h. Amplifications and restriction digestions were performed on a T100 Thermal Cycler (Bio-Rad, Singapore). PCR and RFLP products were separated in 1–3.4% agarose gels, prepared in 1 × TAE buffer, depending on the length of analyzed DNA fragments, and visualized in a ChemiDoc XRS imaging system (Bio-Rad, Hercules, CA, USA). SNP characteristics, primer sequences, and details of the PCR-RFLP assays are presented in Table 2. Figure S1 shows the PCR-RFLP profiles obtained for *ANGPT2* rs3020221, *FLT1* rs722503, *TLR2* rs3804099 and *TLR9* rs187084 polymorphisms by electrophoresis in 2.5% agarose gels.

### 2.4. Statistical Analysis

Clinical data were compared between the PTL and control groups of the examined pregnant women, using the *t*-test or the Mann–Whitney U test for quantitative variables, and by the Pearson chi-square test for qualitative features. The offspring of the pregnant women were compared using the Mann–Whitney U or the Pearson chi-square test, depending on analyzed data. Differences in Apgar between 1 and 5 min were determined among the PTLs, the controls and all the pregnant women in the study, using the Wilcoxon signed-rank test. The APTT and PLT parameters were compared between the studied groups of the patients using the *t*-test or the Mann–Whitney U test. The distribution of genotypes and alleles of the studied polymorphisms, as well as the relationships of genotypes with PTL were determined using the SNPStats software [48]. All the genotypes were described in Hardy–Weinberg (H-W) equilibrium categories. Logistic regression analyses were performed to determine inheritance models for the associations of genotypes with PTL and to demonstrate a possible effect of the selected coagulation parameters and clinical features on the disease. The best inheritance models were selected using the Akaike information criterion (AIC). Both crude and adjusted analyses were performed to show the relationship between genetic changes in the studied polymorphisms and PTL. The allele distribution was compared between the examined patient groups using the Pearson chi-square test. The relationships for the APTT and PLT parameters, determined between periods from 22 to 35 and from 37 to 41 weeks of pregnancy, were estimated using a paired *t*-test or the Wilcoxon signed-rank test depending on the normality of analyzed parameters. The relationships among various PLT parameters were estimated in all pregnant women using Spearman’s rank correlations. Various statistical analyses were carried out by means of the NCSS 2004 software, including *t*-tests, the Mann–Whitney U test, the Pearson chi-square test, the Wilcoxon signed-rank test, Spearman’s rank correlation and logistic regression models for the contribution of clinical data to PTL. The obtained results were accepted as statistically significant at the significance level of *p* ≤ 0.050.

## 3. Results

### 3.1. Patient Characteristics

The study group of the women with PTL and the control group were similar in terms of age (*p* = 0.052), the rate of primiparity (*p* = 1.000) and mode of delivery (*p* = 0.759; see Table 1). The gestational age at delivery was significantly lower among the PTL women when compared to the control patients (*p* = 0.004). Considering the offspring of the examined women, the fetal weight and neonatal Apgar in 1 and 5 min were comparable between the studied groups (*p* = 0.068, *p* = 0.471 and *p* = 0.854, respectively; see Table 1). Significant differences in Apgar between 1 and 5 min were determined among the PTLs, the controls and all the examined pregnant women (*p* ≤ 0.001). Male newborns were significantly more prevalent among the PTLs when compared to the control mothers (*p* = 0.045). Among the examined pregnant women, the APTT and PLT parameters, including PDW and MPV, reached similar values between 22 and 35 (*p* = 0.345, *p* = 0.337 and *p* = 0.453, respectively), as well as between 37 and 41 weeks of gestation (*p* = 0.089, *p* = 0.070 and *p* = 0.076, respectively; see Table 1). Regarding the PLT count and PCT determined between 22 and 35 weeks of pregnancy, significantly lower values were found in the PTLs than in the controls (*p* = 0.013 and *p* = 0.022, respectively). However, the parameters were comparable in the pregnant women between 37 and 41 weeks of gestation (*p* = 0.616 and *p* = 0.978, respectively). Considering the disorders identified before 35 weeks of ongoing pregnancy, anemia and vaginal bleeding were significantly more common among the patients with PTL than in the controls (*p* ≤ 0.001 and *p* = 0.011, respectively). In turn, gestational DM (GDM) and hypertension were more frequently identified among the control women than in the PTL patients (*p* = 0.006 and *p* = 0.024, respectively). In the logistic regression model, both anemia and bleeding were associated with an increased risk of PTL (OR 5.48, 95% CI 2.21–13.55, *p* ≤ 0.001 and OR 7.46, 95% CI 1.43–39.03, *p* = 0.017, respectively), while the prevalence of GDM was reduced in the patients with the disease (OR 0.11, 95% CI 0.02–0.55, *p* = 0.007). In the control pregnant women, neither the risk of miscarriage nor PTL had been identified in their previous pregnancies (see Table 1).

### 3.2. APTT and PLT Parameters in Pregnant Women

All the studied pregnant women reached the reference ranges for APTT and PLT counts. Among the PTLs, the controls and all the pregnant women, significant differences were observed in PLT counts and in PDW and MPV parameters between the periods from 22 to 35 and from 37 to 41 weeks of pregnancy (*p* ≤ 0.050; see Supplementary Table S1). In turn, the APTT and PCT values were similar between the studied pregnancy periods. Considering all the pregnant women, the PLT counts were negatively correlated with PDW and MPV values (*p* ≤ 0.001; see Supplementary Table S2), while they were positively associated with PCT (*p* ≤ 0.001). A positive correlation was also established between PDW and MPV parameters (*p* ≤ 0.001). However, no correlation was found between PDW and PCT or between MPV and PCT parameters.

### 3.3. Hardy-Weinberg Equilibrium

Among all the studied pregnant women, genotypes in the polymorphisms of *ANGPT2*, *FLT1*, *TLR2*, *TLR6* and *TLR9* genes were found in the H-W equilibrium (*p* > 0.050). Considering *CSF2* SNP, the H-W equilibrium was preserved in the PTL patients (*p* = 0.420), while the deviation was significant in the control group (*p* = 0.039).

### 3.4. Associations of Genotypes and Alleles with PTL

**Figure 1:**
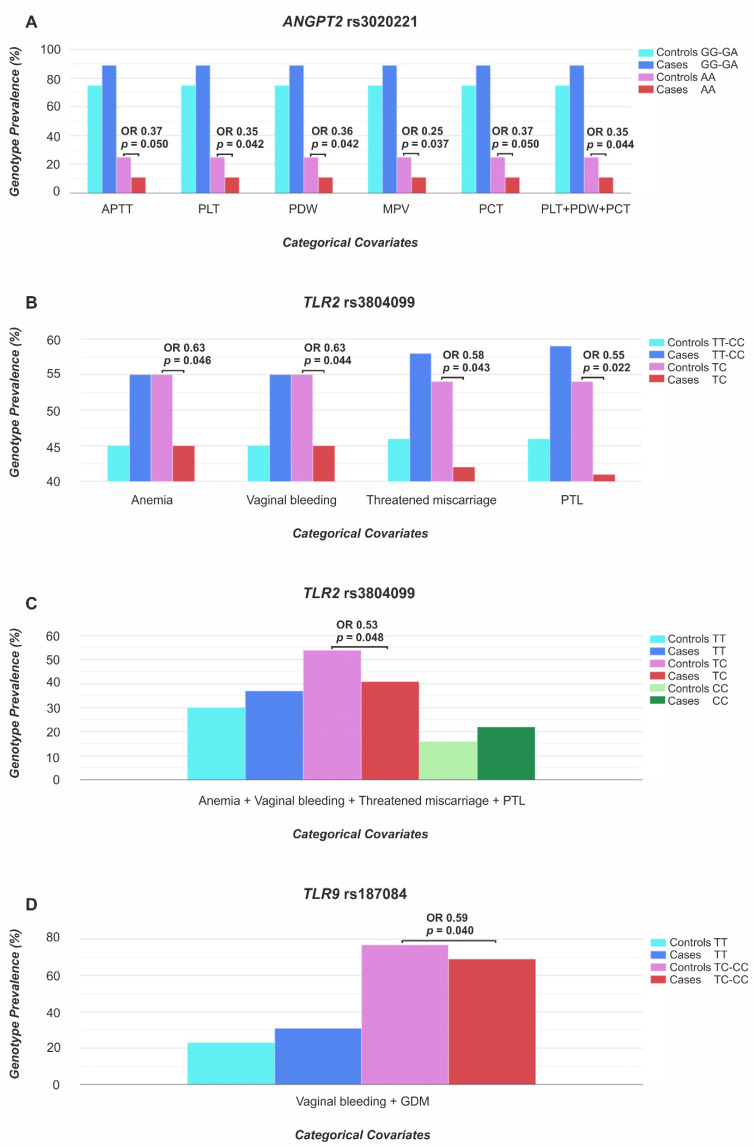
Associations of *ANGPT2* rs3020221 (**A**), *TLR2* rs3804099 (**B**,**C**) and *TLR9* rs187084 (**D**) genotypes with PTL, adjusted by pregnancy-affecting factors. The categorical covariates in the pregnancies, current at that time, were the following APTT and PLT parameters: the PLT count, PDW, MPV and PCT and pregnancy complications, including: anemia, GDM and vaginal bleeding, while previous pregnancy disorders included threatened miscarriage and PTL. OR, odds ratio; *p* ≤ 0.050 was considered significant; APTT, activated partial thromboplastin time; PLT, platelet; PDW, PLT distribution width; MPV, mean PLT volume; PCT, plateletcrit; PTL, spontaneous preterm labor; GDM, gestational diabetes mellitus; AA, CC, GA, GG, TC, TT: genotypes in the analyzed polymorphisms.

Both genotypes and alleles within the studied polymorphisms of the *ANGPT2*, *CSF2* and *FLT1*, the *TLR2*, *TLR6* and *TLR9* genes were similarly distributed between the PTLs and the control pregnant women (Supplementary Tables S3 and S4). Considering *ANGPT2* rs3020221, a further analysis, adjusted by APTT, as well as PLT parameters, determined between 22 and 35 weeks of ongoing pregnancy, showed a significantly lower prevalence of AA homozygotes among the PTLs when compared to the control pregnant women in recessive models (Table 3). The results, corrected simultaneously by the PLT counts and by PDW and PCT values, also showed a significant association of the AA homozygous status in rs3020221 with PTL in the recessive model (OR 0.35, 95% CI 0.13–0.93, *p* = 0.044). In the case of *TLR2* rs3804099, the analysis, adjusted by anemia or vaginal bleeding, observed between 22 and 35 weeks of ongoing pregnancy, identified a significant relationship between TC heterozygotes and PTL in over-dominant models (OR 0.63, 95% CI 0.40–0.99; *p* = 0.046 and 0.044, respectively; Table 3). A correction of the results for threatened miscarriage or PTL observed in previous pregnancies also showed a significant association of TC heterozygotes in rs3804099 with PTL in over-dominant models (Table 3). The adjustment of genotype profiles in *TLR2* rs3804099, simultaneously by anemia and bleeding in ongoing pregnancy, and a threatened miscarriage or PTL, identified in previous pregnancies, also showed a relationship between TC heterozygotes and PTL in the over-dominant model (OR 0.51, 95% CI 0.30–0.88, *p* = 0.014, see Table 4). Considering the *TLR9* rs187084, results adjusted by vaginal bleeding and GDM reported between 22 and 35 weeks of ongoing pregnancy showed TC and CC genotypes significantly less frequently among the PTLs than among the control pregnant women in the dominant model (OR 0.59, 95% CI 0.35–0.98, *p* = 0.040, see Table 3). The most important results regarding the associations of genotypes in *ANGPT2* rs3020221, *TLR2* rs3804099 and *TLR9* rs187084 with PTL, adjusted for the selected factors and influencing the course of pregnancy, are presented in Figure 1.

### 3.5. Sample Size Calculation

Considering the allele frequencies identified for the polymorphisms analyzed in this study, the minimum sample size should be 175 pregnant women, with a 95% confidence level and a 5% margin of error. The value was obtained in relation to the results for *TLR9* rs187084.

## 4. Discussion

The reported study demonstrated that *ANGPT2* rs3020221 minor AA homozygotes were significantly less common in women with PTL than in the control group, as the results were adjusted for the APTT and PLT parameters between 22 and 35 weeks of gestation. Thus far, hypercoagulation has been suggested as the main PTL-related factor [34].

Both the intrinsic and extrinsic coagulation pathways were found to be activated in the PTL [34]. PLT activation was also higher in pregnancies complicated by PE and FGR than in normal gestation and in non-pregnant women [49,50,51,52]. Due to the significant role of the previously described coagulation changes in the development of both normal pregnancy and PTL, the adjustment of current results for the APTT and PLT parameters seems important. Our research demonstrated a significantly higher PLT count, observed between 22 and 35 weeks of gestation, compared to 37 to 41 weeks of pregnancy in all the groups of pregnant women, which was a typical change associated with the development of pregnancy. Moreover, a significantly lower PLT count and PCT were observed in the women with PTL between 22 and 35 weeks of gestation, compared to the controls. All the groups of pregnant women presented significantly higher values of both PDW and MPV determined between the 37th and 41st week of pregnancy when compared to those identified between the 22nd to 35th weeks of gestation. The changes in PDW and MPV levels appeared to reflect the compensatory increases associated with dilutional thrombocytopenia during pregnancy [53]. In our study, an inverse relationship between the PLT count and MPV was also previously shown in pregnant women, revealing pregnancy as a state of compensated thrombocytolysis [54].

In the case of *TLR2* rs3804099, we found TC heterozygotes to be associated with PTL when adjusted for anemia and vaginal bleeding, observed between 22 and 35 weeks in ongoing pregnancies, as well as for threatened miscarriage or PTL from previous pregnancies. Several studies have shown anemia to contribute to an increased risk of adverse effects in both mother and newborn, including PTD, SGA, postpartum hemorrhage, PE, low APGAR score and neonatal death [55,56,57,58,59,60]. According to the meta-analysis, reported in 2019, the risk of pregnancy disorders was approximately two to three times higher in women with anemia [61]. Therefore, it seems necessary to adjust the results, obtained in a current cohort of pregnant women, in terms of anemia as an important risk factor of pregnancy disorders. In our study, anemia was significantly more common in the women with PTL when compared to the full-term control group. Similarly, vaginal bleeding was significantly more frequent in the women with PTL when compared to the control subjects. Previously, it was reported that vaginal bleeding during the first and second trimesters of pregnancy had contributed to the risk of preterm birth associated with ultrasound cervical length [62]. It is noteworthy that physiological vaginal bleeding is observed in the event of a miscarriage, as well as labor-related cervical change, then termed as a “bloody show” usually preceding labor [63]. Therefore, the bleeding episodes found in our study are more common in the women with PTL and may have also been due to cervical remodeling rather than to a bleeding disorder and, hence, not influenced by changes, either in PT or APTT or PLT counts. As for threatened miscarriage, it has previously been shown to be associated with an increased rate of late pregnancy and perinatal complications, including PTD, pPROM, placenta previa, pregnancy-induced hypertension/PE, low birth weight and neonatal admission to intensive care units [64,65].

Ex-vivo studies, conducted on microvascular endothelial cells and rheumatoid arthritis (RA) explants of whole synovial tissue have shown that TLR2 induces angiogenic tube formation and ANGPT2 expression [66]. In the case of *ANGPT2* rs3020221 polymorphism, the A allele has previously been shown to be related to unsuccessful in vitro fertilization [23]. Considering *TLR2* rs3804099, the C allele has been found to be associated with an increased production of several cytokines, including IL10, IL8 and TNF-alpha, in peripheral blood leukocytes, following LPS stimuli [67]. However, conflicting results were obtained regarding the involvement of the T allele of rs3804099 in PTB [68]. In Latin Americans and Tibetans, the T allele correlated with an increased risk of PTB, while in the Iranian population the tested allele was involved in disease resistance [69,70,71]. To date, rs3804099 has also been associated with gastric cancer, hepatocellular carcinoma, and papillary thyroid cancer [72,73,74]. Among patients with colon cancer, the CT or TT genotypes in tested *TLR2* SNP were correlated with a 45% or 38% increase of disease risk, respectively [75].

The present study of the Polish pregnant women suggests that AA homozygotes in *ANGPT2* rs3020221 and TC heterozygotes in *TLR2* rs3804099 possibly play a protective role against PTL. Moreover, both rs3020221 and rs3804099 may be associated with altered coagulation related to PTL due to the involvement of ANGPT2 in the prothrombotic pathways and TLR2 in the prothrombotic PLT function, respectively [76,77]. Decreased thrombus growth was previously determined in germfree and *TLR2* knockout mice when compared to conventionally raised controls, following an injury of the carotid artery [78]. Both TLR2 and TLR6 were also reported to be necessary for the activation of human and murine PLTs by oxidized phospholipids (oxPCCD36), using in vitro methods, as well as genetic deficiency of MyD88 or TLRs in murine PLTs [79]. To date, however, no animal models of PTL have been developed to determine the possible impact of PLT TLR2. Considering *TLR9* rs187084, we found that TC heterozygotes and CC minor homozygotes were significantly less common in the women with PTL when compared to the controls after the adjustment for vaginal bleeding and GDM, which were determined between 22 and 35 weeks of gestation. TLRs have been reported to contribute to a number of autoimmune diseases, including experimental autoimmune encephalomyelitis, systemic lupus erythematosus, RA and type 1 DM (T1DM) [80,81,82,83,84,85]. TLR9-deficient non-obese diabetic mice have been found to be protected against T1DM by impaired IFN-alpha production in pancreatic lymph nodes, and elevated CD73+ T cell expression in peripheral lymph nodes [86,87,88]. TLR9 deficiency used to correlate with pancreatic islet development and beta cell differentiation, which promoted glucose tolerance, increased insulin sensitivity and first-phase insulin secretory response [89]. In turn, TC and CC genotypes in *TLR9* rs5743836 were found to be associated with a 20-fold increased risk of diabetic foot in patients with type 2 DM [90]. Therefore, it seems necessary to correct the genetic results for *TLR9* rs187084 obtained in the present study for GDM.

The CT and TT genotypes within *TLR9* rs187084 were previously reported to be correlated with an increased risk of cervical cancer, while the CT variant was shown to be protective against severe bronchiolitis [91,92,93]. A meta-analysis performed by means of RevMan v.5.3 and Stata v.12.0 showed that the C allele of the rs187084 polymorphism was also associated with an increased risk of cervical cancer [93]. In turn, the T allele was reported as positively correlated with the susceptibility to RA in studied Caucasian women [94]. Moreover, in patients with cervicitis, TC heterozygotes for rs187084 were significantly more frequent when compared to the controls [95]. Thus far, *TLR9* rs187084 has been suggested to create a Sp1 binding site that may be functionally important [96]. The rs187084 C allele was associated with higher *TLR9* transcriptional activity and increased gene expression in Nawalma R20 B cells and peripheral blood mononuclear cells (PBMCs) [32,97]. In addition, the C allele was also correlated with significantly reduced expression levels of inflammatory cytokines, IFN-gamma and TNF-alpha, in PBMCs, compared to the T allele [97]. Similar to FLT1, TLR9 has previously been shown to inhibit angiogenesis. In the case of PTL, *TLR9* rs187084 may be correlated with angiogenesis induction due to lowered TLR9 levels when compared to term pregnant women. Additionally, rs187084 may contribute to PTL-related coagulation changes through decreased *TLR9* transcription in PLTs [98,99]. We also suggest a possible influence of *TLR2* rs3804099 and *TLR9* rs187084 on ANGPT2 prothrombotic activity in PTL, as both TLR2 and TLR9 were previously reported to have affected the ANGPT2 levels in human microvascular endothelial cells and ex-vivo RA synovial explants, as well as in HPMEC, respectively [19,66]. The current research on human and murine PLTs has also shown that the TLR9/MyD88 pathway is involved in PLT activation, granule secretion and aggregation in vitro, as well as in vivo thrombosis, after the induction by carboxyalkylpyrrole protein adducts [99]. In turn, thrombin, a PLT agonist, has been found to increase TLR9 expression in human PLTs, suggesting an intracellular localization of the receptor [100]. However, as with TLR2, no study has been reported on the effect of PLT TLR9 on PTL in any of the animal models of the disease. Future research is expected to further investigate the genetic basis of the mechanisms involved in angiogenesis as well as in coagulation driving PTL.

In a genetic association study, it is very important to enroll a group of patients with a disease, isolated for a particular study and without other accompanying disorders. We have ruled out inter alia, women with pPROM or cervical insufficiency both from the PTL and control cohorts studied, all of which can be considered to be the strengths of our research. Among the various risk factors of PTL, pPROM is one of the most serious pregnancy complications, causing one-third of all PTLs and found in approximately 3–4% of all deliveries [101]. Cervical insufficiency occurs in 0.05 to 2.0% of pregnant women and is a well-known risk factor for PTD and mid-trimester pregnancy loss [102]. Therefore, it was extremely important to exclude women with pPROM or cervical insufficiency from the PTL group in order to determine the genetic background of the studied pregnancy disorder, which was not affected by other related risk factors.

With the study’s limitations in mind, it should be noted that the included cases and the controls were not matched in terms of gestational age. Ideally, the pregnant women in the control group should have been from the 22nd week of pregnancy, just as the enrolled women with PTL. However, healthy pregnant women are usually hospitalized at PMMH-RI from 37 weeks of gestation and for delivery only. In contrast, not all pregnant women admitted to the department before the 37th week of gestation decide to give birth at PMMH-RI. Therefore, it would have been difficult to assemble a sufficiently large control group of pregnant women at the time of the study, who would have been monitored at PMMH-RI from 22 weeks of gestation.

Despite the limitation of our research, its outcomes reveal new and significant data on the possible association of selected SNPs with PTL. Previously, these reported genetic changes were not detected in PTL nor in large-scale genetic analyses.

## 5. Conclusions

The present research showed that three polymorphisms from angiogenesis-related genes—*ANGPT2* rs3020221, *TLR2* rs3804099 and *TLR9* rs187084—are significantly associated with PTL when the outcomes are adjusted for the factors influencing the normal development of pregnancy. The findings are both novel and important for a better understanding of the mechanisms involved in PTL. These results may be useful in risk stratification procedures, regarding the women susceptible to PTL, to ensure rapid intervention and/or provide high-risk care in time.

## Figures and Tables

**Table 1:** Characteristics of the women with spontaneous preterm labor and the controls, included into the study.

		Controls	Cases	*p*-Value ^a^
Number		160	160	
Age (years)		29.04 ± 4.98	27.97 ± 4.83	0.052
Primiparous women, n ^b^ (%)		96 (60.0%)	97 (60.6%)	1.000
Current pregnancy disorders, n (%)	Anemia	7 (4.4%)	29 (18.1%)	≤0.001
	GDM ^c^	12 (7.5%)	2 (1.3%)	0.006
	Hypertension	5 (3.1%)	0 (0.0%)	0.024
	Vaginal bleeding	2 (1.3%)	11 (6.9%)	0.011
Previous pregnancy disorders, n (%)	Threatened miscarriage	0/123 (0.0%)	19/128 (14.8%)	≤0.001
	PTL ^d^	0/123 (0.0%)	7/125 (5.6%)	0.008
APTT (s) ^e^	22–35 weeks of pregnancy	27.4 (24.0–32.6)	27.85 (22.9–36.7)	0.345
	37–41 weeks of pregnancy	28.23 ± 2.24	27.78 ± 2.20	0.089
Platelet parameters	22–35 weeks of pregnancy:			
	No. [×10^9^/L] ^f^	240 (164–324)	220 (125–387)	0.013
	PDW (fL) ^g^	12.5 (8.8–16.5)	12.55 (9.3–20.3)	0.337
	MPV (fL) ^h^	10.7 (8.8–12.1)	10.65 (9.1–14.2)	0.453
	PCT (%) ^i^	0.25 (0.16–0.35)	0.23 (0.14–0.39)	0.022
	37–41 weeks of pregnancy:			
	No. [×10^9^/L]	213 (151–398)	215 (144–326)	0.616
	PDW (fL)	13.7 (9.0–23.7)	14.1 (9.7–19.3)	0.070
	MPV (fL)	11.18 ± 0.96	11.38 ± 0.98	0.076
	PCT (%)	0.24 (0.16–0.40)	0.24 (0.16–0.34)	0.978
Delivery	Weeks of pregnancy	40 (37–41)	39 (33–41)	0.004
	Vaginal, n (%)	73 (45.6%)	33 (47.8%)	0.759
	C-section ^j^, n (%)	87 (54.4%)	36 (52.2%)	
Fetal sex, n (%)	Female	81 (50.6%)	25 (36.2%)	0.045
	Male	79 (49.4%)	44 (63.8%)	
Newborn data	Weight (percentiles)	74.5 (10–100)	66 (5–100)	0.068
	Apgar in 1 min	10 (7–10)	10 (6–10)	0.471
	Apgar in 5 min	10 (7–10)	10 (7–10)	0.854

^a^ *p*-value, *p* ≤ 0.050 is considered significant; ^b^ n, number; ^c^ GDM, gestational diabetes mellitus; ^d^ PTL, spontaneous preterm labor; ^e^ APTT [s], activated partial thromboplastin time [second]; ^f^ No., platelet count; ^g^ PDW, platelet distribution width; ^h^ MPV, mean platelet volume; ^i^ PCT, plateletcrit; ^j^ C-section, caesarean section.

**Table 2:** PCR-RFLP assays, used in the genotyping of six SNPs, located in the *ANGPT2*, *CSF2*, *FLT1*, *TLR2*, *TLR6* and *TLR9* genes [25,42,43,44,45,46,47].

Gene	SNP ^a^	MAF ^b^	Primer Sequences (5′-3′)	Restriction Enzyme	Genotypes [bp ^c^]	Agarose Gel [%]
*ANGPT2*	rs3020221	38.5	F: CATTAGAATAGCCTTCAC	Eco57I	CC: 193, 142	2.5
			R: GAGTGTTTACTGACTAAAGG		CT: 335, 193, 142	
					TT: 335	
*CSF2*	rs25882	20.7	F: AAACTTCCTGTGCAACCGA	Alw26I	TT: 110, 46	3.4
			R: TTTCATGAGAGAGCAGCTCCC		TC: 110, 88, 46, 22	
					CC: 88, 46, 22	
*FLT1*	rs722503	25.2	F: TCCGCCTGCATTTTGAACAACTAAGTAG	AvaII	CC: 199, 169	2.5
			R: GGTCTCCTTGGTATTCAAGCACACGTAA		CT: 368, 199, 169	
					TT: 368	
*TLR2*	rs3804099	44.1	F: TTTATCGTCTTCCTGGTTC	MaeII	TT: 361	2.5
			R: CAAATCAGTATCTCGCAGTT		TC: 361, 258, 103	
					CC: 258, 103	
*TLR6*	rs5743810	41.2	F: CTAGTTTATTCGCTATCCAAG	AvaII	AA: 309	2.5
			R: TTGTCAATGCTTTCAATGTCG		AG: 309, 183, 126	
					GG: 183, 126	
*TLR9*	rs187084	40.6	F: CCTGCCTGCCATGATACCAC	AflII	AA: 242, 79	2.5
			R: TGCTAGCACACCGGATCATT		AG: 321, 242, 79	
					GG: 321	

^a^ SNP, single nucleotide polymorphism; ^b^ MAF, minor allele frequency; ^c^ bp, base pair.

**Table 3:** Association of *ANGPT2*, *TLR2* and *TLR9* SNPs with PTL, corrected for APTT and PLT parameters and the occurrence of pregnancy disorders.

Polymorphism	Categorical Covariate		Genetic Model	Genotype	Genotype Prevalence, n ^a^ (%)		OR ^b^ (95 % CI ^c^)	*p*-Value ^d^	AIC ^e^
					Controls	Cases			
*ANGPT2*	Parameters determined from 22 to 35 weeks of current pregnancy	APTT ^f^	Recessive	GG-GA	24 (75.0%)	137 (89.0%)	1.00	0.050	172.7
rs3020221				AA	8 (25.0%)	17 (11.0%)	0.37 (0.14–0.96)		
		PLT ^g^	Recessive	GG-GA	24 (75.0%)	143 (89.4%)	1.00	0.042	169.7
				AA	8 (25.0%)	17 (10.6%)	0.35 (0.13–0.93)		
		PDW ^h^	Recessive	GG-GA	24 (75.0%)	141 (89.2%)	1.00	0.042	173.7
				AA	8 (25.0%)	17 (10.8%)	0.36 (0.14–0.92)		
		MPV ^i^	Recessive	GG-GA	24 (75.0%)	141 (89.2%)	1.00	0.037	211.9
				AA	8 (25.0%)	17 (10.8%)	0.25 (0.07–0.92)		
		PCT ^j^	Recessive	GG-GA	24 (75.0%)	141 (89.2%)	1.00	0.050	170.6
				AA	8 (25.0%)	17 (10.8%)	0.37 (0.14–0.96)		
		PLT + PDW + PCT	Recessive	GG-GA	24 (75.0%)	141 (89.2%)	1.00	0.044	173
				AA	8 (25.0%)	17 (10.8%)	0.35 (0.13–0.93)		
*TLR2*	Current pregnancy disorders	Anemia	Over-dominant	TT-CC	72 (45.0%)	88 (55.0%)	1.00	0.046	429.5
rs3804099				TC	88 (55.0%)	72 (45.0%)	0.63 (0.40–0.99)		
		Vaginal bleeding	Over-dominant	TT-CC	72 (45.0%)	88 (55.0%)	1.00	0.044	438.4
				TC	88 (55.0%)	72 (45.0%)	0.63 (0.40–0.99)		
	Previous pregnancy disorders	Threatened miscarriage	Over-dominant	TT-CC	57 (46.3%)	74 (57.8%)	1.00	0.043	322.7
				TC	66 (53.7%)	54 (42.2%)	0.58 (0.35–0.98)		
		PTL ^k^	Over-dominant	TT-CC	57 (46.3%)	74 (59.2%)	1.00	0.022	334.8
				TC	66 (53.7%)	51 (40.8%)	0.55 (0.33–0.92)		
*TLR9*	Current pregnancy disorders	Vaginal bleeding + GDM ^l^	Dominant	TT	37 (23.1%)	50 (31.2%)	1.00	0.040	431.1
rs187084				TC-CC	123 (76.9%)	110 (68.8%)	0.59 (0.35–0.98)		

^a^ n, number; ^b^ OR, odds ratio; ^c^ 95% CI, confidence interval; ^d^ *p*-value, *p* ≤ 0.050 is considered significant; ^e^ AIC, Akaike information criterion; ^f^ APTT, activated partial thromboplastin time; ^g^ PLT, platelet; ^h^ PDW, PLT distribution width; ^i^ MPV, mean PLT volume; ^j^ PCT, plateletcrit; ^k^ PTL, spontaneous preterm labor; ^l^ GDM, gestational diabetes mellitus.

**Table 4:** Relationship between *TLR2* rs3804099 and spontaneous preterm labor, adjusted for anemia and vaginal bleeding in the current pregnancy, and for threatened miscarriage or PTL in previous pregnancies.

Genetic Model	Genotype	Genotype Prevalence, n ^a^ (%)		OR ^b^ (95 % CI ^c^)	*p*-Value ^d^	AIC ^e^
		Controls	Cases			
Codominant	TT	37 (30.1%)	46 (37.1%)	1.00	0.048	313.8
	TC	66 (53.7%)	51 (41.1%)	0.53 (0.29–0.97)		
	CC	20 (16.3%)	27 (21.8%)	1.10 (0.52–2.33)		
Dominant	TT	37 (30.1%)	46 (37.1%)	1.00	0.140	315.8
	TC-CC	86 (69.9%)	78 (62.9%)	0.66 (0.37–1.16)		
Recessive	TT-TC	103 (83.7%)	97 (78.2%)	1.00	0.180	316.1
	CC	20 (16.3%)	27 (21.8%)	1.58 (0.81–3.09)		
Over-dominant	TT-CC	57 (46.3%)	73 (58.9%)	1.00	0.014	311.9
	TC	66 (53.7%)	51 (41.1%)	0.51 (0.30–0.88)		

^a^ n, number; ^b^ OR, odds ratio; ^c^ 95% CI, confidence interval; ^d^ *p*-value, *p* ≤ 0.050 is considered significant; ^e^ AIC, Akaike information criterion.

## Data Availability

The data presented in this study are available on request from the corresponding author.

## Supplementary Materials

Supplementary materials can be found at https://www.mdpi.com/article/10.3390/cimb44070203/s1.
