# Supplementary material for: Association of Single Nucleotide Polymorphisms from Angiogenesis-Related Genes, ANGPT2, TLR2 and TLR9, with Spontaneous Preterm Labor
Source: Curr Issues Mol Biol. 2022 Jun 30;44(7):2939–55. doi: 10.3390/cimb44070203 (PMC9322696; doi:10.3390/cimb44070203)
Supplement: Supplementary file 1 [file cimb-44-00203-s001.zip › cimb-1752256-Supplementary tables.pdf]

# Association of Single Nucleotide Polymorphisms from Angiogenesis-Related Genes, *ANGPT2*, *TLR2* and *TLR9,* with Spontaneous Preterm Labor

**Table S1.** APTT and PLT parameters during the development of pregnancy.

| Parameters | Pregnancy period (weeks) | | *p*-value [a] |
|---|---|---|---|
| | 22-35 | 37-41 | |
| **Controls** | | | |
| **APTT** [b] | 27.77 ± 2.14 | 27.89 ± 2.17 | 0.290 |
| **PLT** [c] | 240 (164-324) | 213 (151-398) | 0.018 |
| **PDW** [d] | 12.57 ± 2.05 | 13.73 ± 1.96 | ≤ 0.001 |
| **MPV** [e] | 10.66 ± 0.89 | 11.11 ± 0.90 | ≤ 0.001 |
| **PCT** [f] | 0.26 ± 0.05 | 0.26 ± 0.05 | 1.000 |
| **PTL** [g] **cases** | | | |
| **APTT** | 27.83 ± 2.45 | 27.78 ± 2.24 | 0.393 |
| **PLT** | 220 (125-387) | 215 (144-326) | 0.006 |
| **PDW** | 12.55 (9.3-20.3) | 14.1 (9.7-19.3) | ≤ 0.001 |
| **MPV** | 10.81 ± 0.79 | 11.36 ± 0.98 | ≤ 0.001 |
| **PCT** | 0.23 (0.14-0.39) | 0.24 (0.16-0.34) | 0.974 |
| **All pregnant women** | | | |
| **APTT** | 27.81 ± 2.34 | 27.82 ± 2.20 | 0.483 |
| **PLT** | 232.41 ± 42.60 | 221.43 ± 45.62 | ≤ 0.001 |
| **PDW** | 12.55 (8.8-20.3) | 13.9 (9.0-23.7) | ≤ 0.001 |
| **MPV** | 10.76 ± 0.83 | 11.27 ± 0.96 | ≤ 0.001 |
| **PCT** | 0.24 (0.14-0.39) | 0.24 (0.16-0.40) | 0.969 |

[a] *p*-value, $p \leq 0.050$ is considered significant; [b] APTT, activated partial thromboplastin time; [c] PLT, platelet; [d] PDW, PLT distribution width; [e] MPV, mean PLT volume; [f] PCT, plateletcrit; [g] PTL, spontaneous preterm labor.

**Table S2.** Correlations among PLT parameters in the pregnant women.

| PLT [a] parameters | R [b] | *t*-value | *p*-value [c] |
|---|---|---|---|
| **Between 22 and 35 weeks of pregnancy** | | | |
| **PLT & PDW** [d] | -0.3373 | 4.912 | ≤ 0.001 |
| **PLT & MPV** [e] | -0.3739 | 5.439 | ≤ 0.001 |
| **PLT & PCT** [f] | 0.8693 | 24.117 | ≤ 0.001 |
| **PDW & MPV** | 0.9419 | 37.820 | ≤ 0.001 |
| **PDW & PCT** | 0.0362 | 0.497 | 0.620 |
| **MPV & PCT** | -0.0071 | 0.096 | 0.923 |
| **Between 37 and 41 weeks of pregnancy** | | | |
| **PLT & PDW** | -0.3555 | 5.680 | ≤ 0.001 |
| **PLT & MPV** | -0.3821 | 6.161 | ≤ 0.001 |
| **PLT & PCT** | 0.9118 | 33.165 | ≤ 0.001 |
| **PDW & MPV** | 0.9673 | 56.802 | ≤ 0.001 |
| **PDW & PCT** | -0.0175 | 0.261 | 0.794 |
| **MPV & PCT** | -0.0278 | 0.415 | 0.679 |

[a] PLT, platelet; [b] R, Spearman rank correlation coefficient; [c] *p*-value, $p \leq 0.050$ is considered significant; [d] PDW, PLT distribution width; [e] MPV, mean PLT volume; [f] PCT, plateletcrit.

**Table S3.** Distribution of genotypes in *ANGPT2*, *CSF2*, *FLT1*, *TLR2*, *TLR6* and *TLR9* genes between the women with PTL and the controls.

| Polymorphism | Genetic model | Genotype | Genotype prevalence, n [a] (%) | | OR [b] (95 % CI [c]) | *p*-value [d] | AIC [e] |
|---|---|---|---|---|---|---|---|
| | | | Controls | Cases | | | |
| ***ANGPT2* rs3020221** | Codominant | GG | 65 (40.6%) | 64 (40.0%) | 1.00 | 0.640 | 448.7 |
| | | GA | 73 (45.6%) | 79 (49.4%) | 1.10 (0.69-1.76) | | |
| | | AA | 22 (13.8%) | 17 (10.6%) | 0.78 (0.38-1.61) | | |
| | Dominant | GG | 65 (40.6%) | 64 (40.0%) | 1.00 | 0.910 | 447.6 |
| | | GA-AA | 95 (59.4%) | 96 (60.0%) | 1.03 (0.66-1.60) | | |
| | Recessive | GG-GA | 138 (86.2%) | 143 (89.4%) | 1.00 | 0.390 | 446.9 |
| | | AA | 22 (13.8%) | 17 (10.6%) | 0.75 (0.38-1.46) | | |
| | Over-dominant | GG-AA | 87 (54.4%) | 81 (50.6%) | 1.00 | 0.500 | 447.2 |
| | | GA | 73 (45.6%) | 79 (49.4%) | 1.16 (0.75-1.80) | | |
| ***CSF2* rs25882** | Codominant | TT | 109 (68.1%) | 109 (68.1%) | 1.00 | 0.730 | 449.0 |
| | | TC | 41 (25.6%) | 44 (27.5%) | 1.07 (0.65-1.77) | | |
| | | CC | 10 (6.2%) | 7 (4.4%) | 0.70 (0.26-1.91) | | |
| | Dominant | TT | 109 (68.1%) | 109 (68.1%) | 1.00 | 1.000 | 447.6 |
| | | TC-CC | 51 (31.9%) | 51 (31.9%) | 1.00 (0.62-1.60) | | |
| | Recessive | TT-TC | 150 (93.8%) | 153 (95.6%) | 1.00 | 0.450 | 447.1 |
| | | CC | 10 (6.2%) | 7 (4.4%) | 0.69 (0.25-1.85) | | |
| | Over-dominant | TT-CC | 119 (74.4%) | 116 (72.5%) | 1.00 | 0.700 | 447.5 |
| | | TC | 41 (25.6%) | 44 (27.5%) | 1.10 (0.67-1.81) | | |
| ***FLT1* rs722503** | Codominant | TT | 96 (60.0%) | 94 (58.8%) | 1.00 | 0.380 | 447.7 |
| | | CT | 53 (33.1%) | 60 (37.5%) | 1.16 (0.73-1.84) | | |
| | | CC | 11 (6.9%) | 6 (3.8%) | 0.56 (0.20-1.57) | | |
| | Dominant | TT | 96 (60.0%) | 94 (58.8%) | 1.00 | 0.820 | 447.6 |

| | | | | | | | |
|---|---|---|---|---|---|---|---|
| | | CT-CC | 64 (40.0%) | 66 (41.2%) | 1.05 (0.67-1.65) | | |
| | Recessive | TT-CT | 149 (93.1%) | 154 (96.2%) | 1.00 | 0.210 | 446.0 |
| | | CC | 11 (6.9%) | 6 (3.8%) | 0.53 (0.19-1.46) | | |
| | Over-dominant | TT-CC | 107 (66.9%) | 100 (62.5%) | 1.00 | 0.410 | 446.9 |
| | | CT | 53 (33.1%) | 60 (37.5%) | 1.21 (0.77-1.92) | | |
| ***TLR2* rs3804099** | Codominant | TT | 48 (30.0%) | 56 (35.0%) | 1.00 | 0.190 | 446.2 |
| | | TC | 88 (55.0%) | 72 (45.0%) | 0.70 (0.43-1.15) | | |
| | | CC | 24 (15.0%) | 32 (20.0%) | 1.14 (0.59-2.20) | | |
| | Dominant | TT | 48 (30.0%) | 56 (35.0%) | 1.00 | 0.340 | 446.7 |
| | | TC-CC | 112 (70.0%) | 104 (65.0%) | 0.80 (0.50-1.27) | | |
| | Recessive | TT-TC | 136 (85.0%) | 128 (80.0%) | 1.00 | 0.240 | 446.2 |
| | | CC | 24 (15.0%) | 32 (20.0%) | 1.42 (0.79-2.53) | | |
| | Over-dominant | TT-CC | 72 (45.0%) | 88 (55.0%) | 1.00 | 0.073 | 444.4 |
| | | TC | 88 (55.0%) | 72 (45.0%) | 0.67 (0.43-1.04) | | |
| ***TLR6* rs5743810** | Codominant | CC | 62 (38.8%) | 59 (36.9%) | 1.00 | 0.350 | 447.5 |
| | | CT | 80 (50.0%) | 74 (46.2%) | 0.97 (0.60-1.57) | | |
| | | TT | 18 (11.2%) | 27 (16.9%) | 1.58 (0.79-3.16) | | |
| | Dominant | CC | 62 (38.8%) | 59 (36.9%) | 1.00 | 0.730 | 447.5 |
| | | CT-TT | 98 (61.2%) | 101 (63.1%) | 1.08 (0.69-1.70) | | |
| | Recessive | CC-CT | 142 (88.8%) | 133 (83.1%) | 1.00 | 0.150 | 445.5 |
| | | TT | 18 (11.2%) | 27 (16.9%) | 1.60 (0.84-3.04) | | |
| | Over-dominant | CC-TT | 80 (50.0%) | 86 (53.8%) | 1.00 | 0.500 | 447.2 |
| | | CT | 80 (50.0%) | 74 (46.2%) | 0.86 (0.55-1.33) | | |
| ***TLR9* rs187084** | Codominant | TT | 37 (23.1%) | 50 (31.2%) | 1.00 | 0.230 | 446.6 |
| | | TC | 89 (55.6%) | 76 (47.5%) | 0.63 (0.37-1.07) | | |
| | | CC | 34 (21.2%) | 34 (21.2%) | 0.74 (0.39-1.40) | | |
| | Dominant | TT | 37 (23.1%) | 50 (31.2%) | 1.00 | 0.100 | 444.9 |
| | | TC-CC | 123 (76.9%) | 110 (68.8%) | 0.66 (0.40-1.09) | | |

| | | | | | | |
|---|---|---|---|---|---|---|
| Recessive | TT-TC | 126 (78.8%) | 126 (78.8%) | 1.00 | NA [f] | 447.6 |
| | CC | 34 (21.2%) | 34 (21.2%) | 1.00 (0.59-1.71) | | |
| Over-dominant | TT-CC | 71 (44.4%) | 84 (52.5%) | 1.00 | 0.150 | 445.5 |
| | TC | 89 (55.6%) | 76 (47.5%) | 0.72 (0.46-1.12) | | |

[a] n, number; [b] OR, odds ratio; [c] 95% CI, confidence interval; [d] *p*-value, $p \leq 0.050$ is considered significant; [e] AIC, Akaike information criterion; [f] NA, not analyzed.

**Table S4.** The frequency of alleles from angiogenesis-related genes in the women with spontaneous preterm labor and the controls.

| Polymorphism | Allele | No. [a] of alleles (%) | | Chi-Square | $p$-value [b] |
|---|---|---|---|---|---|
| | | Controls | Cases | | |
| ***ANGPT2*** | A | 117 (36.6%) | 113 (35.3%) | 0.109 | 0.742 |
| **rs3020221** | G | 203 (63.4%) | 207 (64.7%) | | |
| ***CSF2*** | C | 61 (19.1%) | 58 (18.1%) | 0.093 | 0.761 |
| **rs25882** | T | 259 (80.9%) | 262 (81.9%) | | |
| ***FLT1*** | C | 75 (23.4%) | 72 (22.5%) | 0.079 | 0.778 |
| **rs722503** | T | 245 (76.6%) | 248 (77.5%) | | |
| ***TLR2*** | C | 136 (42.5%) | 136 (42.5%) | 0.000 | 1.000 |
| **rs3804099** | T | 184 (57.5%) | 184 (57.5%) | | |
| ***TLR6*** | C | 204 (63.8%) | 192 (60.0%) | 0.954 | 0.329 |
| **rs5743810** | T | 116 (36.2%) | 128 (40.0%) | | |
| ***TLR9*** | C | 157 (49.1%) | 144 (45.0%) | 1.060 | 0.303 |
| **rs187084** | T | 163 (50.9%) | 176 (55.0%) | | |

[a] No., number; [b] $p$-value, $P \leq 0.050$ is considered significant.
