# Supplementary figures and images for: Association of Single Nucleotide Polymorphisms from Angiogenesis-Related Genes, ANGPT2, TLR2 and TLR9, with Spontaneous Preterm Labor
Source: Curr Issues Mol Biol. 2022 Jun 30;44(7):2939–55. doi: 10.3390/cimb44070203 (PMC9322696; doi:10.3390/cimb44070203)

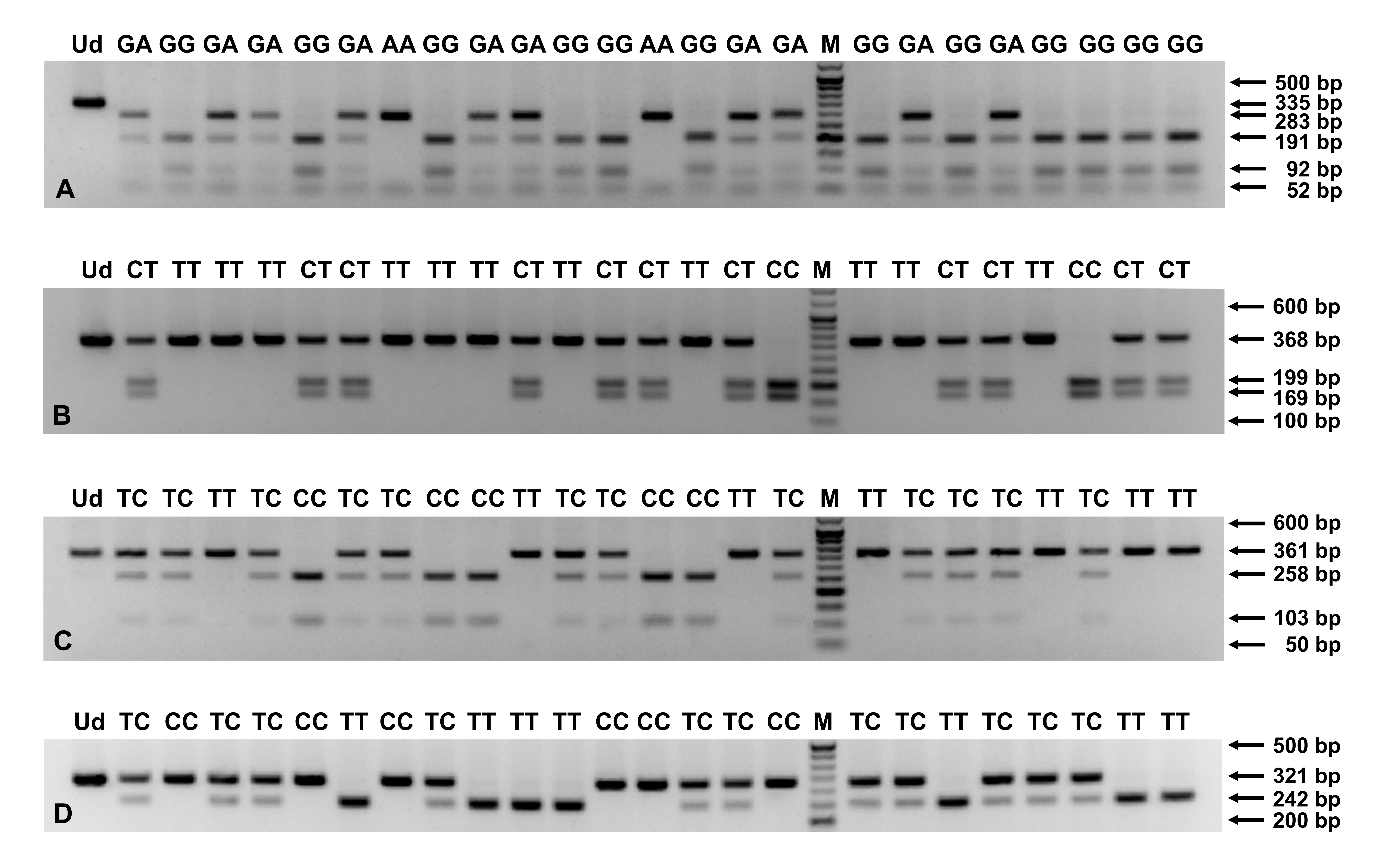

Supplement: Supplementary file 1 [file cimb-44-00203-s001.zip › Figure S1.tif]
